# Incidence and risk factors for developing infection in patients presenting with uninfected diabetic foot ulcers

**DOI:** 10.1371/journal.pone.0177916

**Published:** 2017-05-17

**Authors:** Limin Jia, Christina N. Parker, Tony J. Parker, Ewan M. Kinnear, Patrick H. Derhy, Ann M. Alvarado, Flavia Huygens, Peter A. Lazzarini, on behalf of the Diabetic Foot Working Group, Queensland Statewide Diabetes Clinical Network (Australia)

**Affiliations:** 1 Institute of Health and Biomedical Innovation, Queensland University of Technology, Brisbane, Queensland, Australia; 2 Department of Endocrinology, Ningxia People’s Hospital, Yinchuan, Ningxia Hui Autonomous Region, China; 3 School of Nursing, Queensland University of Technology, Brisbane, Queensland, Australia; 4 School of Biomedical Sciences, Queensland University of Technology, Brisbane, Queensland, Australia; 5 Allied Health Research Collaborative, Metro North Hospital & Health Service, Brisbane, Queensland, Australia; 6 Clinical Access and Redesign Unit, Queensland Health, Brisbane, Queensland, Australia; 7 School of Clinical Sciences, Queensland University of Technology, Brisbane, Queensland, Australia; University of Illinois at Urbana-Champaign, UNITED STATES

## Abstract

**Objective:**

There is a paucity of research on patients presenting with uninfected diabetic foot ulcers (DFU) that go on to develop infection. We aimed to investigate the incidence and risk factors for developing infection in a large regional cohort of patients presenting with uninfected DFUs.

**Methods:**

We performed a secondary analysis of data collected from a validated prospective state-wide clinical diabetic foot database in Queensland (Australia). Patients presenting for their first visit with an uninfected DFU to a Diabetic Foot Service in one of thirteen Queensland regions between January 2012 and December 2013 were included. Socio-demographic, medical history, foot disease history, DFU characteristics and treatment variables were captured at the first visit. Patients were followed until their DFU healed, or if their DFU did not heal for 12-months, to determine if they developed a foot infection in that period.

**Results:**

Overall, 853 patients were included; mean(standard deviation) age 62.9(12.8) years, 68.0% male, 90.9% type 2 diabetes, 13.6% indigenous Australians. Foot infection developed in 342 patients for an overall incidence of 40.1%; 32.4% incidence in DFUs healed <3 months, 55.9% in DFUs healed between 3–12 months (*p*<0.05). Independent risk factors (Odds Ratio (95% confidence interval)) for developing infection were: DFUs healed between 3–12 months (2.3 (1.6–3.3)), deep DFUs (2.2 (1.2–3.9)), peripheral neuropathy (1.8 (1.1–2.9)), previous DFU history (1.7 (1.2–2.4)), foot deformity (1.4 (1.0–2.0)), female gender (1.5 (1.1–2.1)) and years of age (0.98 (0.97–0.99)) (all *p*<0.05).

**Conclusions:**

A considerable proportion of patients presenting with an uninfected DFU will develop an infection prior to healing. To prevent infection clinicians treating patients with uninfected DFUs should be particularly vigilant with those presenting with deep DFUs, previous DFU history, peripheral neuropathy, foot deformity, younger age, female gender and DFUs that have not healed by 3 months after presentation.

## Introduction

Diabetic foot infections are a well-recognised risk factor for hospitalisation and amputation [1–5]. According to a recent meta-analysis one in every 30 hospitalised patients at any given time is affected by a diabetic foot infection [6]. Additionally, patients with diabetes who develop an infection have been reported to have a 155-fold increased risk of amputation compared to those who do not [5]. Nearly all diabetic foot infections originate in a diabetic foot ulcer (DFU) [3–6] and the prevalence of these infections in DFUs have been reported to range between 25–60% [3–5, 7–10]. Although the critical nature and prevalence of infected DFUs are well appreciated [1–10], the development of these infections in the first place has received less attention [5, 11].

One prospective study of 1,666 diabetes patients reported a 9% incidence for developing a foot infection over a two-year follow up period [5]. Furthermore, the same study indicated a 61% incidence for developing a foot infection over a similar two-year period in a subgroup of 247 patients with a DFU; however, this subgroup included patients presenting with a mix of uninfected and infected DFUs [5]. Similarly, a small number of studies have investigated the risk factors for developing a diabetic foot infection and report patients presenting with peripheral neuropathy, peripheral arterial disease, previous DFUs and deep DFUs are more likely to develop diabetic foot infections [5, 11]. However, to our knowledge no study has specifically investigated the incidence and risk factors for developing an infection in a population of patients presenting with an uninfected DFU. Thus, the primary aim of this study was to investigate the incidence and risk factors for developing an infection in patients presenting with an uninfected DFU. A secondary aim was to investigate the incidence of infection in patients with different DFU types and different DFU healing time durations.

## Materials and methods

### Study design

This study was a secondary analysis of data collected from a validated prospective state-wide clinical diabetic foot database in Queensland, Australia (Queensland High Risk Foot Form Database [12, 13]). The study received multi-site ethical approvals from two Australian Human Research Ethics Committees (HRECs); The Prince Charles Hospital (HREC/15/QPCH/177) and the Queensland University of Technology (1500000700). Furthermore, the study received legal approvals from the Queensland Statewide Diabetes Clinical Network Data Access committee and a Queensland Public Health Act 2005 waiver (QCHO/009321/RD006012) to use confidential de-identified information from the database for the purposes of this study. Thus, individual consent was not required or available for this study.

### Settings and participants

Queensland is the third largest Australian state in terms of population, second largest in area and the most decentralised state with extremely diverse demography and geography [14]. Patients attending an outpatient Diabetic Foot Service in 13 of the 17 Hospital and Health Service areas in Queensland for treatment have their diabetic foot clinical data captured at each visit on a validated Queensland High Risk Foot Form (QHRFF) [12, 13, 15]. The QHRFF data is then collated and cleaned in the centralised QHRFF database for DFU healing and recurrence clinical benchmarking and research purposes [12, 13].

Eligible patients for this study were patients attending an aforementioned Diabetic Foot Service in Queensland for their first clinical visit that recorded an uninfected DFU between 1^st^ January 2012 and 31^st^ December 2013. The first clinical visit was defined as the first date the patient attended the Diabetic Foot Service between 1^st^ January 2012 and 31^st^ December 2013. A patient may have attended the service prior to 2012; however, this data was not captured for this study. A DFU was defined as a full thickness wound beneath the ankle on a patient with diabetes [4, 12]. An uninfected DFU was defined according to the International Working Group on the Diabetic Foot classification system as having no clinical signs or symptoms of infection [16, 17]. Patients were then followed from their first clinical visit until their DFU healed or if it did not heal for 12 months. Exclusion criteria included patients who did not have their infection status recorded at their first clinical visit (baseline) or at follow up visits prior to their DFU healing or if it did not heal prior to 12 months (follow up).

### Variables collected

For the purposes of this study the explanatory variables were those collected using the QHRFF at the patient’s first clinical visit. If data were missing for a variable, the second visit’s data was used for that variable(s) if available, provided the second visit was within one month of the first visit. The QHRFF data collection procedures, methods and definitions have been reported in detail elsewhere [12, 13, 18]. In brief, the QHRFF has been reported to be valid and reliable for the capture of multiple self-reported and clinically diagnosed variables when collected by clinicians with a range of diabetic foot disease experience [12].

The self-reported variables included: demographic (age, sex, indigenous status and residential postcode); diabetes history (diabetes type, diabetes duration, glycated haemoglobin (HbA1c) and blood glucose levels (BGLs) >15 mmol/L in the previous 14 days); medical history (hypertension, dyslipidaemia, cardiovascular disease, chronic kidney disease and smoking status); foot disease history (previous foot ulcer and previous amputation); and past foot treatment in the previous 14 days (by podiatrist, general practitioner, surgeon, physician, nurse, orthotists or other) [12]. Patient’s postcode of residence was transformed into the social determinant variables of socioeconomic status (according to the Australian Index of Relative Social Disadvantage [19]) and geographical remoteness status (according to the Accessibility/Remoteness Index of Australia [20]).

The clinically diagnosed variables included: foot risk factors (peripheral neuropathy (PN), lack of protective sensation to a 10-gram monofilament on at least 2 of 3 plantar forefoot locations [21, 22]; peripheral arterial disease (PAD), toe systolic pressure <70 mmHg [21, 22]; foot deformity, scored at least 3 points on a 6-point foot deformity score [22]; suspected acute Charcot foot, red, hot, swollen, unilateral neuropathic foot joint without a DFU near the suspected Charcot joint [22]); foot ulcer characteristics (ulcer surface area (mm^2^); grade and depth, according to the University of Texas Diabetic Wound Classification System [4]; deep ulcers, scored a 2 (“wound penetrating to tendon or capsule”) or 3 (“wound penetrating to bone or joint”) [4]; and infection status according to the International Working Group on the Diabetic Foot classification system [16, 17]) [12]. DFU treatment provided on the first clinical visit was also recorded, including if the DFU was treated with: sharp debridement; appropriate wound dressings; prescribed antibiotics; optimum offloading in a cast walker; appropriate footwear; and patient education on DFU care [12, 22]. Lastly, foot ulcer healing time was captured and defined by subtracting the date of first clinical visit (as defined above) from the date the ulcer was recorded as healed (complete epithelialisation) [4, 12]. Ulcer healing time was categorised into: i) healed <3 months (<90 days since first visit), ii) healed between 3–12 months (91–365 days), iii) not healed at 12 months (ulcer had not healed at 365 days since first clinical visit).

The primary outcome variable for this study was the development of a foot infection prior to when the DFU healed or if the DFU did not heal then prior to 12-months after the first clinical visit. Foot infection was defined according to the International Working Group on the Diabetic Foot classification system as at least two clinical signs or symptoms of infection in or around the DFU including purulence, erythema, pain, tenderness, warmth and/or induration [16, 17, 23]. Patients were also sub-grouped into the following types of DFU: neuropathic (PN and no PAD), ischaemic (PAD and no PN), neuro-ischaemic (PN and PAD), post-surgical (recent non-healed minor amputation procedure regardless of PN or PAD), other (none of the aforementioned DFU types) or unknown (PN, PAD or post-surgical was not recorded). For patients with multiple DFUs a combined surface area of the multiple DFU was calculated, and the DFU type, grade, depth and treatment characteristics for the worst DFU used [12].

### Statistical analyses

Statistical analyses were performed with IBM SPSS 23.0 Statistics for Windows (SPSS Inc., Chicago, IL, USA) or GraphPad Software. Categorical variables were expressed as proportions (%) and continuous variables were expressed as a mean (standard deviation (SD)). Incidence was expressed as the proportion (%) of patients developing an infection of eligible patients; for overall patients, different DFU types, and ulcer healing time categories. For categorical variables Pearson’s chi-squared tests were used to test for any differences between groups (*p*<0.05) and Fisher’s exact test with Bonferroni corrections were used for post-hoc pairwise comparisons (*p*<0.005) [24, 25]. For continuous variables analysis of variance (ANOVA) with Tukey’s post-hoc test used were used to test for differences between groups (*p*<0.05) [24, 25]. Univariate logistic regression analyses were used to test for crude associations (*p*<0.1) [26]. All variables achieving a crude association were included in a multivariate logistic regression analysis to test for independent risk factors [26]. A backwards stepwise method was used to remove non-significant variables (*p*>0.05) at each step until only variables reaching statistical significance remained (*p*<0.05) [26, 27]. If collinearity was identified using a correlation matrix (>0.9) the variables with the lowest odds ratio was excluded [26]. Hosmer and Lemeshow goodness of fit, Omnibus degrees of freedom, Negelkerke pseudo R^2^ tests and significance were assessed at each step [26, 27]. Missing data were treated by excluding cases with missing data as the proportion of missing data were <5% in the model [26].

## Results

Overall, 922 patients were eligible for inclusion in this study. Of these 69 (7.5%) patients were excluded: 53 (5.7%) because their infection status was not recorded at baseline and 16 (1.7%) because their infection status was not recorded on follow up. The remaining 853 included patients had a mean age (SD) of 62.9 (12.8) years, 68.0% were male, 13.6% were indigenous and 90.9% had type 2 diabetes. After the 12 month follow up period 454 (53.2%) patients’ DFUs healed <3 months, 222 (26.0%) healed between 3–12 months and 177 (20.8%) had not healed at 12 months.

Table 1 displays the patient characteristics for the different DFU types: 316 (37.0%) had neuropathic, 242 (28.4%) neuro-ischemic, 53 (6.2%) ischemic, 68 (8.0%) post-surgical, 41 (4.8%) other and 133 (15.6%) unknown DFU types. Differences between patients with different DFU types included: ischaemic ulcer patients were of older age (*p*<0.05), more had hypertension and cardiovascular disease, and fewer had previous foot ulcers (all *p*<0.005); neuro-ischaemic ulcer patients were also of older age (*p*<0.05), more had chronic kidney disease, previous amputations and past foot treatment from an orthotist (all *p*<0.005); post-surgical ulcer patients had larger ulcer surface areas (*p*<0.05) and more had previous amputations (*p*<0.005); other ulcer patients had fewer males, foot deformities, optimum offloading treatment and appropriate footwear treatment (all *p*<0.005); and, patients with unknown ulcer types had fewer BGLs >15 mmol/L, hypertension, dyslipidaemia, CVD and smokers, and more had previous foot ulcers (all *p*<0.005). Although no differences existed for diabetes duration and HbA1c, it is noted that data on these two variables were missing in >50% of patients.

**Table 1:** Participant characteristics for each diabetic foot ulcer type (number (%) unless otherwise stated).

	n	All	Neuropathic	Ischaemic	Neuro-Ischaemic	Post-Surgical	Other	Unknown	*p* Value
**Patients**	853	853	316	53	242	68	41	133	
**Demographics**									
Age years (SD)	853	62.9(12.8)	59.2(12.6)	68.7(9.8)**	67.4(11.4)**	60.2(12.0)	58.7(14.7)	63.6(12.8)	<0.001*
Age range years		27–92	28–89	48–90	27–90	40–92	27–87	32–92	
Male sex	846	575 (68.0%)	211 (67.2%)	35 (66.0%)	172 (71.1%)	54 (79.4%)	19 (48.7%)**	84 (64.6%)	0.027*
Indigenous	521	71 (13.6%)	30 (13.4%)	2 (5.9%)	21 (12.7%)	8 (14.0%)	5 (18.5%)	5(35.7%)	0.145
**Social Determinants**									
Socioeconomic Status	846	846	314	51	240	68	41	132	
Most disadvantaged		199 (23.5%)	88 (28.0%)	8 (15.7%)	58 (24.2%)	10 (14.7%)	8 (19.5%)	27 (20.5%)	
Second most disadvantaged		188 (22.2%)	63 (20.1%)	14 (27.5%)	51 (21.3%)	14 (20.6%)	11 (26.8%)	35 (26.5%)	
Middle		130 (15.4%)	41 (13.1%)	8 (15.7%)	40 (16.7%)	15 (22.1%)	4 (9.8%)	22 (16.7%)	
Second least disadvantaged		241 (28.5%)	83 (26.4%)	21 (41.2%)	71 (29.6%)	17 (25.0%)	16 (39.0%)	33 (25.0)	
Least disadvantaged		88 (10.4%)	39 (12.4%)	0	20 (8.3%)	12 (17.6%)	2 (4.9%)	15 (11.4%)	0.497
Geographic Remoteness	846	846	314	51	240	68	41	132	
Major city		373 (44.1%)	144 (45.9%)	18 (35.3%)	93 (38.8%)	33 (48.5%)	17 (41.5%)	68 (51.5%)	
Inner regional area		165 (19.5%)	57 (18.2%)	13 (25.5%)	50 (20.8%)	14 (20.6%)	9 (22.0%)	22 (16.7%)	
Outer regional area		194 (22.9%)	71 (22.6%)	14 (27.5%)	60 (25.0%)	13 (19.1%)	12 (29.3%)	24 (18.2%)	
Remote area		66 (7.8%)	16 (5.1%)	4 (7.8%)	25 (10.4%)	5 (7.4%)	3 (7.3%)	13 (9.8%)	
Very remote area		48 (5.7%)	26 (8.3%)	2 (3.9%)	12 (5.0%)	3 (4.4%)	0	5 (3.8%)	0.261
**Diabetes History**									
Type 2 diabetes	853	775 (90.9%)	283 (89.6%)	51 (96.2%)	224 (92.6%)	62 (91.2%)	37 (90.2%)	118 (88.7%)	0.537
Duration years (SD)	365	19.7(11.0)	19.1(9.4)	19.1(12.4)	21.9(12.5)	17.0(10.6)	14.6(11.7)	20.6(7.3)	0.056
HbA1c % (SD)	220	8.4(2.0)	8.7(2.1)	7.3(1.4)	8.3(2.0)	8.4(1.7)	8.8(3.1)	7.8(1.8)	0.270
HbA1c mmol/mol ^a^	220	68	72	56	67	68	73	62	0.270
BGL >15mmol/L	762	130 (17.1%)	62 (22.1%)	8 (16.0%)	33 (14.7%)	9 (15.0%)	9 (25.0%)	9 (8.1%)**	0.015*
**Medical History**									
Hypertension	853	211 (24.7%)	88 (27.8%)	29 (54.7%)**	53 (21.9%)	18 (26.5%)	17 (41.5%)	6 (4.5%)**	<0.001*
Dyslipidaemia	853	139 (16.3%)	54 (17.1%)	10 (18.9%)	51 (21.1%)	12 (17.6%)	7 (17.1%)	5 (3.8%)**	0.001*
CVD	853	90 (10.6%)	24 (7.6%)	18 (34.0%)**	37 (15.3%)	6 (8.8%)	4 (9.8%)	1 (0.8%)**	<0.001*
CKD ^b^	853	60 (7.0%)	22 (7.0%)	5 (9.4%)	29 (12.0%)**	1 (1.5%)	0	3 (2.3%)	0.001*
Smoker	853	37 (4.3%)	19 (6.0%)	3 (5.7%)	7 (2.9%)	5 (7.4%)	3 (7.3%)	0**	0.035*
**Past Foot Treatment**									
Podiatrist	853	800 (93.8%)	293 (92.7%)	51 (96.2%)	227 (93.8%)	60 (88.2%)	40 (100%)	128 (96.2%)	0.115
GP	853	20 (2.3%)	10 (3.2%)	1 (1.9%)	5 (2.1%)	2 (2.9%)	1 (2.4%)	1 (0.8%)	0.755
Surgeon	853	14 (1.6%)	8 (2.5%)	0	3 (1.2%)	2 (2.9%)	0	1 (0.8%)	0.450
Physician	853	21 (2.5%)	14 (4.4%)	0	6 (2.5%)	0	0	1 (0.8%)	0.055
Nurse	853	53 (6.2%)	18 (5.7%)	4 (7.5%)	18 (7.4%)	7 (10.3%)	2 (4.9%)	4 (3.0%)	0.375
Orthotist	853	8 (0.9%)	1 (0.3%)	0	7 (2.9%)**	0	0	0	0.015*
Other	853	20 (2.3%)	10 (3.2%)	1 (1.9%)	9 (3.7%)	0	0	0	0.118
**Foot Disease History**									
Previous foot ulcer	853	595 (69.8%)	202 (63.9%)	30 (56.6%)**	184 (76.0%)	46 (67.6%)	26 (63.4%)	107 (80.5%)**	0.001*
Previous amputation	852	242 (28.4%)	74 (23.5%)	5 (9.4%)	83 (34.3%)**	40 (58.8%)**	7 (17.1%)	33 (24.8%)	<0.001*
**Foot Risk Factors**									
Peripheral Neuropathy	726	617 (85.0%)	316 (100%)	0**	242 (100.0%)	53 (80.3%)	0**	6 (75.0%)	<0.001*
PAD	720	330 (45.8%)	0**	53 (100.0%)	242 (100.0%)	32 (49.2%)**	0**	3 (100.0%)	<0.001*
Foot deformity	728	460 (63.2%)	209 (66.1%)	24 (45.3%)	159 (66.0%)	47 (72.3%)	13 (32.5%)**	8 (61.5%)	<0.001*
Acute Charcot Foot	723	18 (2.5%)	13 (4.1%)	0	4 (1.7%)	0	1 (2.5%)	0	0.191
**Foot Ulcer Characteristics**									
Ulcer area mm^2^ (SD) ^c^	740	235.7(610.4)	184.7(449.2)	210.5(351.6)	226.3(594.8)	585.8(941.3)**	394.3(1317.7)	163.0(488.2)	<0.001*
Deep ulcer ^d^	843	66 (7.8%)	20 (6.5%)	2 (3.8%)	18 (7.5%)	9 (13.2%)	5 (12.8%)	12 (8.9%)	0.333
Ulcer healing time	853								
Healed <3 months		454 (53.2%)	177 (56.0%)	29 (54.7%)	115 (47.5%)	43 (63.2%)	19 (46.3%)	71 (53.4%)	
Healed 3–12 months		222 (26.0%)	75 (23.7%)	10 (18.9%)	71 (29.3%)	15 (22.1%)	10 (24.4%)	41 (30.8%)	
Not healed at 12 months		177 (20.8%)	64 (20.3%)	14 (26.4%)	56 (23.1%)	10 (14.7%)	12 (29.3%)	21 (15.8%)	0.188
**Treatment**									
Debrided Ulcer	849	765 (90.1%)	295 (93.4%)	45 (86.5%)	206 (86.2%)	61 (89.7%)	35 (85.4%)	123 (92.5%)	0.063
Dressing Appropriate	845	820 (97.0%)	303 (96.5%)	51 (98.1%)	230 (96.2%)	66 (98.5%)	39 (97.5%)	131 (98.5%)	0.766
Antibiotics Prescribed	851	58 (6.8%)	19 (6.0%)	1 (1.9%)	23 (9.6%)	6 (8.8%)	4 (9.8%)	5 (3.8%)	0.146
Offloading Optimum	851	395 (46.4%)	138 (43.9%)	24 (45.3%)	123 (50.8%)	25 (36.8%)	12(29.3%)**	73 (54.9%)	0.016*
Footwear Appropriate	848	505 (59.6%)	206 (65.2%)	31 (58.5%)	131 (54.8%)	36 (53.7%)	17 (42.5%)**	84 (63.2%)	0.024*
Patient Educated	851	843(99.1%)	314 (99.7%)	53 (100%)	238 (98.8%)	67 (98.5%)	39 (95.1%)	132 (99.2%)	0.104
**Foot Infection Incidence**									
All foot ulcer patients	853	342 (40.1%)	133 (42.1%)	14 (26.4%)	106 (43.8%)	22 (32.4%)	13 (31.7%)	54 (40.6%)	0.111
Ulcer healing time	853								
Healed <3 months	454	147 (32.4%)	67 (37.9%)	8 (27.6%)	40 (34.8%)	10 (23.3%)	3 (15.8%)	19 (26.8%)	0.152
Healed 3–12 months	222	124 (55.9%)	40 (53.3%)	4 (40.0%)	41 (57.7%)	8 (53.3%)	5 (50.0%)	26 (63.4%)	0.785
Not healed at 12 months	177	71 (40.1%)	26 (40.6%)	2 (14.3%)	25 (44.6%)	4 (40.0%)	5 (41.7%)	9 (42.9%)	0.487

* *p* < 0.05 for ANOVA (continuous) or Pearson’s chi-squared (categorical)

** Group(s) identified to be different to other groups using *p* < 0.05 for adjusted Post hoc tests (continuous) or *p* < 0.005 Fishers exact test with Bonferroni corrections (categorical)

^a^ HbA1c mmol/mol IFFC converted from HbA1c % NGSP

^b^ CKD is a combination of CKD and ESKD (End Stage Kidney Disease)

^c^ Ulcer area was measured by multiplying the longest edge x the widest edge in mm

^d^ Deep ulcer is an ulcer scoring a 2 or 3 on the University of Texas Diabetic Wound Classification System

BGL>15 mmol/L: Blood Glucose Levels exceeding 15 mmol/L; CKD: Chronic Kidney Disease; CVD: Cardiovascular Disease; GP: General Practitioner; mm^2^: millimetres squared; PAD: Peripheral Arterial Disease; SD: Standard deviation.

Table 1 also shows 342 patients developed a foot infection during follow up for an overall incidence of 40.1%; including 32.4% incidence in DFUs healed <3 month, 55.9% in DFUs healed between 3–12 month and 40.1% in DFUs that had not healed at 12 months (p<0.05). However, there were no differences in foot infection incidence between different DFU types (*p*>0.05).

Table 2 displays the univariate analyses that were conducted on the 720 patients with known DFU types. We excluded the 133 patients with unknown DFU types from the univariate analyses as they recorded large amounts of missing foot-related data, but in the other demographic, social determinant, diabetes history, past foot treatment and treatment variables with limited missing data they reported very few differences compared to known DFU types (*p*>0.05) (Table 1). The variables that achieved a crude univariate association and were included in the multivariate logistic regression model were age, sex, previous foot ulcer, peripheral neuropathy, foot deformity, deep ulcer and ulcer healing time (*p*<0.1).

**Table 2:** Univariate analysis for patients developing an infection of a diabetic foot ulcer (number (%) unless otherwise stated).

	n	All	No Infection	Infection	*p* Value
**Patients**	720	720	432(60.0%)	288(40.0%)	
**Demographics**					
Age years (SD)	720	62.7(12.7)	63.9(12.5)	61.0(12.9)	0.003*
Male sex	716	491 (68.6%)	305 (71.3%)	186 (64.6%)	0.071*
Indigenous	507	66 (13.0%)	38 (13.1%)	28 (12.9%)	1.000
**Social Determinants**					
Socioeconomic Status	714				
Most disadvantaged		172 (24.1%)	105 (24.5%)	67(23.5%)	
Second most disadvantaged		153 (21.4%)	96 (22.4%)	57(20.0%)	
Middle		108 (15.1%)	69 (16.1%)	39(13.7%)	
Second least disadvantaged		208 (29.1%)	112 (26.1%)	96(33.7%)	
Least disadvantaged		73 (10.2%)	47 (11.0%)	26(9.1%)	0.276
Geographic Remoteness	714				
Major city		305 (42.7%)	174 (40.6%)	131(46.0%)	
Inner regional area		143 (20.0%)	86 (20.0%)	57(20.0%)	
Outer regional area		170 (23.8%)	104 (24.2%)	66(23.2%)	
Remote area		53 (7.4%)	37 (8.6%)	16(5.2%)	
Very remote area		43 (6.0%)	28 (6.5%)	15(5.3%)	0.434
**Diabetes History**					
Type 2 diabetes	720	657 (91.3%)	397 (91.9%)	260 (90.3%)	0.536
Duration years (SD)	360	19.7 (11.1)	19.6 (11.3)	19.8(10.8)	0.864
HbA1c % (SD)	205	8.5(2.1)	8.5(2.2)	8.4(1.8)	0.571
HbA1c mmol/mol ^a^	205	69	69	68	0.571
BGL >15mmol/L	651	121 (18.6)	74 (18.9%)	47 (18.1%)	0.895
**Medical History**					
Hypertension	720	205 (28.5%)	131 (30.3%)	74 (25.7%)	0.206
Dyslipidaemia	720	134 (18.6%)	83 (19.2%)	51 (17.7%)	0.681
CVD	720	89 (12.4%)	55 (12.7%)	34 (11.8%)	0.799
CKD ^b^	720	57 (7.9%)	34 (7.9%)	23 (8.0%)	1.000
Smoker	720	37 (5.1%)	21 (4.9%)	16 (5.6%)	0.809
**Past Foot Treatment**					
Yes	720				
Podiatrist	720	672 (93.3%)	402 (93.1%)	270 (93.8%)	0.831
GP	720	19 (2.6%)	12 (2.8%)	7 (2.4%)	0.962
Surgeon	720	13 (1.8%)	10 (2.3%)	3 (1.0%)	0.331
Physician	720	20 (2.8%)	14 (3.2%)	6 (2.1%)	0.487
Nurse	720	49 (6.8%)	26 (6.0%)	23 (8.0%)	0.381
Orthotist	720	8 (1.1%)	5 (1.2%)	3 (1.0%)	1.000
Other	720	20 (2.8%)	12 (2.8%)	8 (2.8%)	1.000
**Foot Disease History**					
Previous foot ulcer	720	488 (67.8%)	270 (62.5%)	218 (75.7%)	<0.001*
Previous amputation	719	209 (29.1%)	115 (26.7%)	94 (32.6%)	0.101
**Foot Risk Factors**					
Peripheral Neuropathy	718	611 (85.1%)	353 (82.1%)	258 (89.6%)	0.008*
PAD	717	327 (45.6%)	194 (45.2%)	133 (46.2%)	0.860
Foot deformity	715	452 (63.2%)	254 (59.2%)	198 (69.2%)	0.008*
Acute Charcot Foot	712	18 (2.5%)	7 (1.6%)	11 (3.9%)	0.108
**Foot Ulcer Characteristics**					
Ulcer area mm^2^ (SD) ^c^	624	249.2(629.9)	234.8(670.0)	270.1(567.2)	0.493
Deep ulcer ^d^	711	55 (7.7%)	22 (5.2%)	33(11.6%)	0.003*
Ulcer healing time	720				
Healed <3 months		383 (53.2%)	255 (59.0%)	128 (44.4%)	
Healed 3–12 months		181 (25.1%)	83 (19.2%)	98 (34.0%)	
Not healed at 12 months		156 (21.7%)	94 (21.8%)	62 (21.5%)	<0.001*
**Treatment**					
Debrided Ulcer	716	642 (89.7%)	379 (88.1%)	263 (92.0%)	0.129
Dressing Appropriate	712	689 (96.8%)	410 (95.8%)	279 (98.2%)	0.112
Antibiotics Prescribed	718	53 (7.4%)	31 (7.2%)	22 (7.7%)	0.927
Offloading Optimum	718	322 (44.8%)	189 (44.0%)	133 (46.2%)	0.609
Footwear Appropriate	715	421 (58.9%)	258 (60.1%)	163 (57.0%)	0.447
Patient Educated	718	711 (99.0%)	427 (99.1%)	284 (99.0%)	1.000

* *p* < 0.1

^a^ HbA1c mmol/mol IFFC converted from HbA1c % NGSP

^b^ CKD is a combination of CKD and ESKD (End Stage Kidney Disease)

^c^ Ulcer area was measured by multiplying the longest edge x the widest edge in mm

^d^ Deep ulcer is an ulcer scoring a 2 or 3 on the University of Texas Diabetic Wound Classification System

BGL>15 mmol/L: Blood Glucose Levels exceeding 15 mmol/L; CKD: Chronic Kidney Disease; CVD: Cardiovascular Disease; GP: General Practitioner; mm^2^: millimetres squared; PAD: Peripheral Arterial Disease; SD: Standard deviation.

Table 3 displays the independent risk factors (Odds Ratio (95% confidence interval)) that predicted infection from the multivariate model and included: ulcers healed between 3–12 months (2.3 (1.6–3.3)), deep ulcers (2.2 (1.2–3.9)), peripheral neuropathy (1.8 (1.1–2.9)), previous foot ulcers (1.7 (1.2–2.4)), foot deformity (1.4 (1.0–2.0)), female gender (1.5 (1.1–2.1)) and years of age (0.98 (0.97–0.99)) (all *p*<0.05). The above multivariate model explained approximately 12.0% of the variance of the infection outcome (Nagelkerke pseudo R^2^ = 0.120; *p*<0.001).

**Table 3:** Independent risk factors for developing an infection of a diabetic foot ulcer.

Risk Factor	Odds Ratio (95% CI)	*p* Value
Age (years)	0.98 (0.97–0.99)	0.002*
Female	1.52 (1.08–2.14)	0.016*
Peripheral Neuropathy	1.77 (1.09–2.86)	0.022*
Foot Deformity	1.44 (1.02–2.04)	0.039*
Previous Foot Ulcer	1.66 (1.16–2.36)	0.005*
Deep Ulcer	2.16 (1.19–3.93)	0.011*
Ulcer healing time		<0.001*
Healed <3 months	Referent	
Healed 3–12 months	2.26 (1.55–3.29)	<0.001*
Not healed at 12 months	1.20 (0.80–1.81)	0.373
*Model Results*:	*Pseudo R*^*2*^: *0*.*120;* *Omnibus*: *df = 8*, *p = <0*.*001*	*Missing*: *17 (2*.*4%);* *H&L*: *p = 0*.*088*

* p < 0.05

df: degrees of freedom; H&L: Hosmer and Lemeshow Goodness of Fit Test; Missing: Excluded missing cases; Omnibus: Omnibus Tests of Model Coefficients; Pseudo R2: Nagelkerke R2.

## Discussion

We found a 40% incidence of developing an infection prior to healing in a large sample of patients presenting with uninfected DFUs across a large representative region of Australia. The incidence increased from 32% in ulcers healed within 3 months to 56% in ulcers healed between 3–12 months. However, there was no statistical differences in the incidence of developing an infection between different types of DFUs. We identified several independent risk factors that predicted infection in patients presenting with an uninfected DFU, including presenting with a deep ulcer, a previous DFU history, peripheral neuropathy, foot deformity, at a younger age and female gender. Furthermore, DFUs that had not healed by 3 months after presentation were also a significant risk factor for infection. This study confirms, that regardless of the type of DFU presenting, a considerable proportion of people with DFUs will develop an infection prior to healing and common risk factors predict these infections.

To our knowledge only a paper by *Lavery and colleagues (2006)* has previously investigated the incidence of diabetic foot infection [5]. *Lavery* reported a 9% incidence of foot infection over a two-year follow up period in 1,666 patients with diabetes who were at different risks of developing DFU (low or high risk) [5]. They also indicated a 61% incidence of infection in a subgroup of 247 patients who developed a DFU (presenting with or without infection) [5]. Our study found a 40% incidence of infection over a one-year follow up period in 853 patients who first presented with an uninfected DFU. Additionally we found the incidence increased from 32% for ulcers healed <3 months to 56% for ulcers healed between 3–12 months; however, the incidence for ulcers not healed at 12 months was no higher than those healed <12 months. Furthermore whilst not ideal, comparing our 32–56% incidence findings to the 26–60% infection prevalence range reported by numerous DFU studies, seems to add further plausibility to our findings [3–5, 7–10]. Overall, interpreting these incidence findings together suggests the chance of developing an infection is double in those with “non-healing ulcers” (>3 months healing time) compared to “healing ulcers” (<3 months) [5, 10].

To our knowledge we are the first to investigate the incidence of developing infection in patients with different types of DFU and interestingly we found no differences. A recent previous study examining the prevalence of infection in different types of DFU also reported no differences [28]. This previous study reported very similar patient characteristics for those patients with different types of DFU to our study which also increases the potential generalisability of our findings [28]. Additionally, our overall DFU patient characteristics were very similar to those reported in other large international DFU studies, which further increases the generalisability of our findings [3, 8–10]. Lastly, our reported socio-demographic characteristics were very similar to the socio-demographic characteristics reported for the general Australian population [14, 19, 20]. Overall, these generalizable findings indicate that all clinicians treating patients with any type of DFU need to be incredibly vigilant to prevent infection and the subsequent significant risk of hospitalisation and amputation [5].

The risk factors we identified for developing foot infection in a large homogenous uninfected DFU population were similar to those identified in other studies investigating more heterogeneous DFU populations [5, 11], with perhaps some novel exceptions. Our identified risk factors with greatest odds of developing infection were highly consistent with these previous studies, including ulcers healed >3months, deep ulcers, peripheral neuropathy, foot deformity and previous ulcer history [5, 11]. It is not surprising that those DFUs that were deepest and had not healed for a longer duration were the leading risk factors for developing soft tissue infection as they expose a greater volume of soft tissue to infective organisms for a longer period of time [5, 29]. Additionally, it is not surprising that DFU patients with peripheral neuropathy and its resultant lack of protective sensation and immunopathy that contribute to delayed local inflammatory responses to infective organisms [30] is also a leading risk factor for developing diabetic foot infection [11]. Whilst foot deformity has not been specifically identified in previous studies, high plantar pressures have been [11] and foot deformity is often used as a surrogate marker of high plantar pressures [22]. Again high plantar pressures, especially in combination with neuropathy and deep ulcers, could be reasonably expected to expose a great volume of soft tissue that has limited inflammatory response to infective organisms [11]. Lastly, ours and previous studies did not capture participant’s history of previous diabetic foot infection [5, 11]; however, considering the high prevalence of infections in DFUs [3–5, 7–10], it is most probable that the identified risk factor of a previous DFU is also surrogate marker of a previous diabetic foot infection history [5, 11]. Thus, it is not surprising that a history of any pathology is a risk factor for future pathology, in this case diabetic foot infection.

The novel risk factors identified in our study were that of younger age and female gender. To our knowledge, age or gender has not been previously identified as a risk factor for soft tissue diabetic foot infection [5, 11]. In contrast to younger age, many studies have reported older age to be a risk factor for developing poor DFU outcomes [28, 31, 32]. However, one large previous study reported an association with younger age in patients with diabetes developing osteomyelitis [33]. Additionally, the mean ages reported from previous studies investigating large populations of DFU patients (approximately 65 years [8, 29, 31]) have generally been older when compared to studies investigating large populations of infected DFU patients (approximately 55 years [11, 33, 34]). It could be hypothesised that younger patients with equivalent DFUs are more physically active for longer durations then their older counterparts which may increase their exposure to infective organisms for longer durations. Also in contrast to female gender, it has been male gender that has been consistently reported to be a risk factor for the development of poor DFU outcomes [11, 29, 31, 32]. However, females have been reported to be at higher risk of other infections in patients with diabetes (such as different urinary tract infections) [35, 36] and perhaps female patients with diabetes are more susceptible to infection in general than males. Interestingly, recently published findings from a large retrospective analysis of outpatients with diabetes in the United States also reports patients with infected DFU were more likely to be younger and female compared to those with uninfected DFU [37]. Overall, these findings suggest the demographic risk factors for developing a DFU may be different to those for developing an infection of a DFU, and thus, more research investigating why these risk factors may be different is recommended

Interestingly, no other characteristics were identified as risk factors for infection in this study. This is somewhat surprising considering other studies have identified PAD in particular to be a risk factor for diabetic foot infection [5, 11]. However, we hypothesise that, due to the heterogeneous populations investigated by these previous studies, PAD was more likely a risk factor for DFU [2–4] rather than diabetic foot infection in someone who has already developed a DFU. We suggest that once PAD has contributed to the development of a DFU, it is then the depth of tissue involvement, the duration of exposure, coupled with the immunopathy and lack of protective sensation from peripheral neuropathy that are the real culprits for developing a foot infection in the first instance [11, 30]. However, we suggest that once a DFU has become infected then underlying PAD may accelerate the progression of the infection and increase the risk of subsequent hospitalisation and amputation [3, 4].

Other large international population-based studies have identified socio-economic status, geographical remoteness and ethnicity to be associated with poor DFU outcomes [38, 39]. Two recent Australian studies also identified specific associations between indigenous Australians and DFU [40, 41]. However, our study which included patients from the diverse socio-demographic, geographical and indigenous backgrounds found in Australia [14, 32] could not find any associations with developing infection. Our findings were consistent with *Peters et al* that could not find any association between socio-demographic factors and diabetic foot infection in the United States [11]. Additionally, they were consistent with a large study by *Holman et al (2015)* investigating DFU healing outcomes across the United Kingdom who also could not find any association with socio-demographic factors [10]. *Holman* hypothesised that the universal best practice standard of DFU care provided to patients attending the services in their study may have counteracted any typical socio-demographic factor influence on negative DFU outcomes [10] and our findings seem to support their hypothesis.

Although, our risk factor findings were relatively consistent with previous studies [5, 11], our study is the first to report the variance predicted from our model containing these risk factors. Collectively our multivariate model explained approximately 12% of the predicted variance for developing infection according to our reported pseudo R^2^. This predicted variance indicates that there must be other risk factors for developing infection in patients with DFU that were not investigated in our study. Thus, we firstly recommended that any future studies investigate the risk factors identified in our study in addition to a range of other diabetes severity, physical activity, plantar pressures, microbial isolates, inflammatory makers and treatment factors in both best practice and standard care settings [42]. Secondly, it is recommended that future studies investigate for any differences in risk factor profiles for developing mild, moderate and severe diabetic foot infection outcomes. Lastly, it is recommended that clinicians managing patients with a DFU should be particularly vigilant in their provision of best practice care if those patients present with a DFU that is deep, have a previous ulcer history, peripheral neuropathy, foot deformity, are of younger age or female. This vigilance should be heightened again if the ulcer has not healed by 3 months. Any improved strategies to prevent infection in patients with DFU should also begin to reduce the significant burden of hospitalisation and amputation in these patients.

### Limitations and strengths

This study had a number of limitations. First, it was secondary analysis of a prospective state-wide clinical diabetic foot database that primarily collects DFU healing and recurrence outcomes for clinical benchmarking and research purposes and not primarily for infection outcomes. However it could be argued that this database’s primary focus on DFU also provides some strength to our study. Second, much missing data was reported in relation to the specific diabetes severity variables (diabetes duration, HbA1c) and for this reason we did not include them in our multivariate models. Therefore, our study was unable to properly investigate if these variables were risk factors for infected DFU. Third, we defined PAD using a toe pressure of <70mmHg, which is arguably a lower threshold than the <0.8 ankle brachial index used by aforementioned studies investigating risk factors for infection [5, 11], and this may have meant we over-reported PAD. Fourth, we excluded patients that did not have infection status recorded at baseline or during follow up; however, the proportion of patients fitting these criteria was small. Fifth, we followed up patients whose DFU had not healed for only 12-months and this may have contributed to an underestimation of our infection incidence for those not healed at 12-months. Sixth, we were only able to obtain dichotomous data reporting if an infection occurred prior to healing and not the date of infection. Thus, like other papers in this field [5, 11] we were able to report incidence rates and perform logistic regression; however, we were unable to report time-to-event survival analyses which may have provided additional more detailed findings. Seventh, although the QHRFF best practice protocol used in this study aligns with national diabetic foot guideline recommendations [22] and recommends at least fortnightly care for DFU patients [12], we can only assume this best practice follow up care was provided. Additionally, we may have missed some infections and other treatments (such as some revascularisation procedures) that were potentially managed elsewhere between visits. Last, no information about microbial isolates or inflammatory markers was collected to determine their association with infection development [42].

This study also had a number of strengths. First, this study’s sample size was the largest of any study longitudinally investigating DFU patients for infection [5, 11], was comparable in sample size to other large longitudinal studies investigating other DFU outcomes [5, 8–10] and had a very high retention rate. Second, the characteristics of included DFU patients and DFU types in our study were generalizable to other regions as we reported very similar characteristics to those reported in other large DFU studies [3, 8–10]. Third, the included patients in our study were representative of the diverse socio-demographic backgrounds reported in the general Australian population [14, 19, 20]. Fourth, all variables were captured on a data collection instrument and database that has been reported to be valid and reliable to collect DFU-related variables, including infection [12, 13, 15]. Fifth, DFU patients were treated according to a best practice protocol on their first visit [12, 22]. Furthermore, this protocol recommends at least fortnightly clinical care and data collection using the QHRFF for DFU patients [22], and thus, it is assumed patients also received frequent best practice care and data collection during follow up [12]. Last, we identified independent risk factors for diabetic foot infection that were adjusted for age, sex and ulcer healing time using a widely recommended multivariate logistic regression analysis model [26, 27], which makes the findings of our study comparable to other similar studies of diabetic foot infection [5, 11]. However, an additional strength of our study’s analysis was that we were the first to report the approximate variance that our identified risk factors collectively predicted for diabetic foot infection.

## Conclusions

This study of a large representative Australian population of patients presenting to Diabetic Foot Services with uninfected diabetic foot ulcers, confirms that a considerable proportion will develop an infection prior to healing, regardless of their ulcer type. Risk factors for developing an infection included initially presenting with a deep ulcer, having had a previous ulcer, having peripheral neuropathy, having a foot deformity, being of younger age and female. In addition an ulcer that had not healed <3 months of this presentation doubled the odds of developing infection. It is recommended that future investigations in this population focus on demographic, diabetes severity, physical activity, microbial isolates and inflammatory marker explanatory variables and different diabetic foot infection severity outcomes. The results of this study should assist to improve risk profiling and treatment of diabetic foot ulcers to prevent future infections.
